# An interesting case of likely *BRCA2* related bilateral breast cancer with metastasis in the fimbrial part of fallopian tube

**DOI:** 10.1186/s13053-020-00139-w

**Published:** 2020-03-19

**Authors:** Lučka Boltežar, Gorana Gašljević, Srdjan Novaković, Vida Stegel, Erik Škof

**Affiliations:** 1 Division of Medical Oncology, Institute of Oncology Ljubljana, Zaloska cesta 2, 1000 Ljubljana, Slovenia; 2 Department of Pathology, Institute of Oncology Ljubljana, Zaloska cesta 2, 1000 Ljubljana, Slovenia; 3 Department of Molecular Diagnostics, Institute of Oncology Ljubljana, Zaloska cesta 2, 1000 Ljubljana, Slovenia

**Keywords:** Breast cancer, *BRCA2*, Oligometastatic, Fimbrial part of fallopian tube, Receptor conversion

## Abstract

**Background:**

In a patient with a germline *BRCA2* pathogenic variant with breast cancer, an adnexal mass can represent either a metachronous primary tumor or a metastasis of the breast cancer. A clear distinction between those two possibilities is crucial since treatments differ substantially and so does survival of the patient.

**Case presentation:**

We present a case of a 47-year-old patient with bilateral breast carcinoma with a germline *BRCA2* pathogenic variant. The first manifestation of the disease was a lump in her left breast in 1998, histological report was invasive ductal carcinoma, triple-negative. She was treated with surgery, chemotherapy and radiotherapy. In 2011 a new occult carcinoma was found in her right axilla, however the specimen was estrogen receptor (ER) and progesterone receptor (PgR) positive. She was treated as a new primary occult carcinoma of the right breast with surgery, radiotherapy and adjuvant hormonal treatment. In 2016 a mass in the left adnexa was found with imaging techniques. She underwent surgery as if it was primary ovarian cancer, yet histology revealed it was a metastasis of a triple-negative breast carcinoma in the fimbrial part of the left Fallopian tube. She received adjuvant chemotherapy after surgery and is now in complete remission.

**Conclusion:**

We present an interesting and quite rare case of two primary breast carcinomas in a patient with a known *BRCA2* pathogenic variant with metastasis in the fimbrial part of the left Fallopian tube. We conclude that there were two primary breast tumours and the one from 2011 spread into the fimbrial part of the left Fallopian tube in 2016. Despite the fact that molecular analyses could not confirm the joint tumour origin, we believe that there was a receptor status conversion over time explaining different receptor status. The possibility of a triple-negative metastasis from the tumour treated in 1998 is less probable. With both of aforementioned possibilities being prognostically unfavourable, the patients’ outcome is so far excellent and she was in complete remission at the time of writing this article.

## Background

Breast is one of the leading cancer sites in females across the globe. It is also the leading cancer site for females in Slovenia [1]. The presence of *BRCA1* or *BRCA2* pathogenic variant poses a significant risk of developing breast and ovarian cancer as well as other types of cancer – gastric, colorectal, uterine cancer, melanoma etc. [2]. Since there is no effective screening method for ovarian cancer so far [2], once identified as a *BRCA* carrier, several preventive measures and implications are suggested by the guidelines [2] for these patients, among which risk-reducing salpingo-oophorectomy is recommended before the age of 40. According to the literature, the so called occult cancers are found in 2–12% when risk-reducing surgery is performed [2, 3].

In a patient with a history of breast cancer with a positive *BRCA1 or 2* pathogenic variant, an adnexal mass can represent either a metachronous primary tumour or a metastasis. Histological examination is necessary. Occult tubo-ovarian cancers are usually smaller and found incidentally in risk-reducing surgery while metastases usually present clinically or are found by imaging techniques, rarely incidentally in the case of prophylactic adnexal removal [3–5]. However, the distinction between the two is clinically important not only from therapeutic, but also from the prognostic point of view: it was shown that if an ovarian mass represents a metastasis of another cancer, the patients’ survival is worse than survival of the patients with primary ovarian cancer [6].

We report a case of a patient with breast cancer with a metastasis into the fimbriae of the left Fallopian tube which was suspected to be a primary ovarian cancer due to her *BRCA2* pathogenic variant.

## Case presentation

**Fig. 1:**
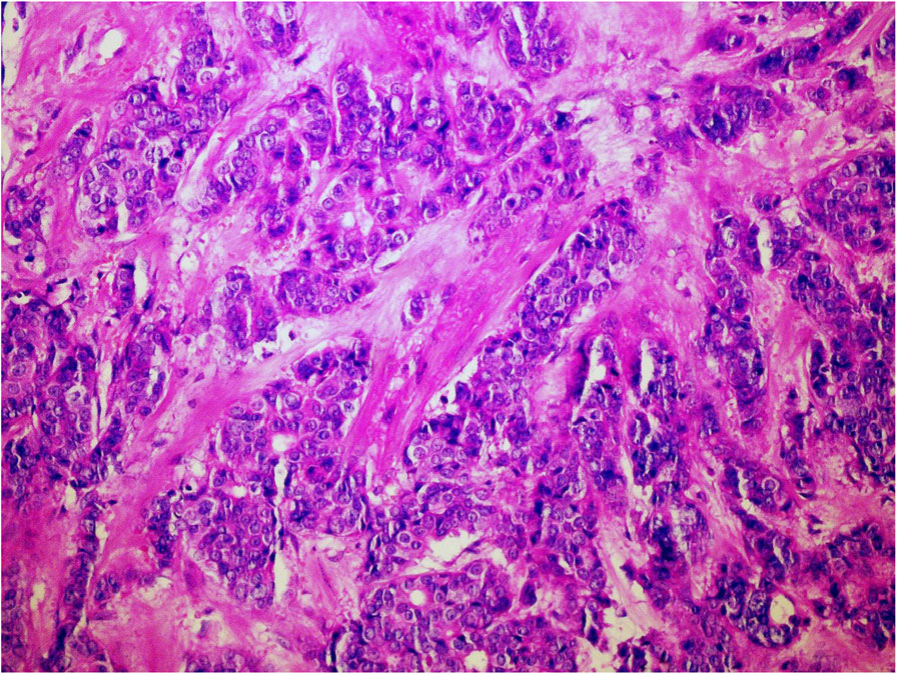
Poorly differentiated invasive duct carcinoma of the left breast; H&E, 20x

A 47-year-old female presented with a lump in her left breast in December 1998. Her family history was unremarkable and her Ca 15–3 level was normal. Tumourectomy was performed in a regional hospital and revealed a poorly differentiated invasive ductal carcinoma measuring 9 mm in the largest diameter (Fig. 1). Oestrogen receptor (ER) and progesterone receptor (PgR) were tested and were negative. Human epidermal growth factor receptor 2 (Her2) status has not been determined yet in those times. She was sent to our Institute for additional treatment. Since pathologist could not have evaluated the status of excisional margins because of the mechanical tissue damage, the quadrectomy and axillar dissection were performed in February 1999. One out of 17 resected lymph nodes was metastatic (1/17) with extracapsular infiltration of perinodal fat tissue while quandractomy specimen revealed only foci of residual ductal carcinoma in situ (DCIS) and reactive changes from the tumourectomy itself. She was treated with adjuvant chemotherapy and irradiation. She received 6 cycles of CMF (cyclophosphamide, methotrexate and fluorouracil) and 50 Gy on her left breast and additional 10 Gy on the tumor bed. The adjuvant treatment was completed in July 1999 and regular follow up was initiated.

**Fig. 2:**
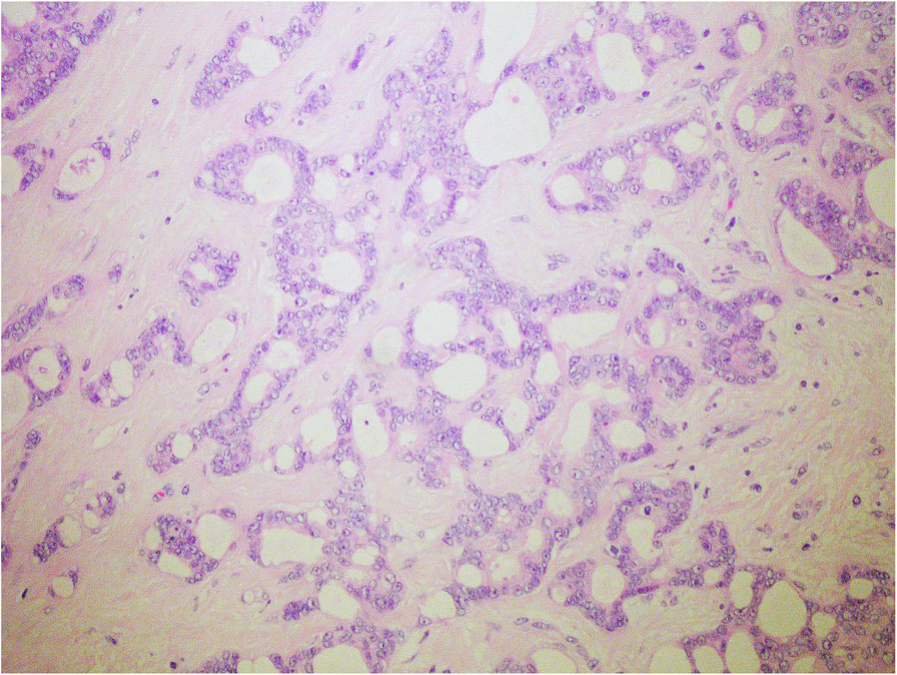
Metastasis of well differentiated invasive carcinoma in the axillar lymph node; H&E, 20x

In September 2011 she had noticed a tumour in her right axilla. An ultrasound of the axillar region revealed 2 × 1 cm pathological lymph node. Cytological examination of the node showed a metastasis of adenocarcinoma. A following magnetic resonance imaging (MRI) of the right breast showed no pathological lesions. Her laboratory blood testing was normal including the Ca 15–3 level, as well as ultrasound of the abdomen and X-ray of the chest. Right axillar dissection was performed in November 2011, 1 out of 19 removed lymph nodes was positive for invasive carcinoma (Fig. 2). This metastasis measured 2 cm in the diameter, extracapsular extension was present. ER was 100% positive and PgR was 90% positive, Her-2 was negative and proliferation index (MIB-1) was 10–15%. Due to the difference of biomarker status of the metastasis in the right axilla and the tumour of the left breast in 1999, she was interpreted to have a new primary, occult cancer of the right breast and was treated with adjuvant hormonal treatment (an aromatase inhibitor) and irradiation of right breast and right axillar region with 50 Gy.

**Fig. 3:**
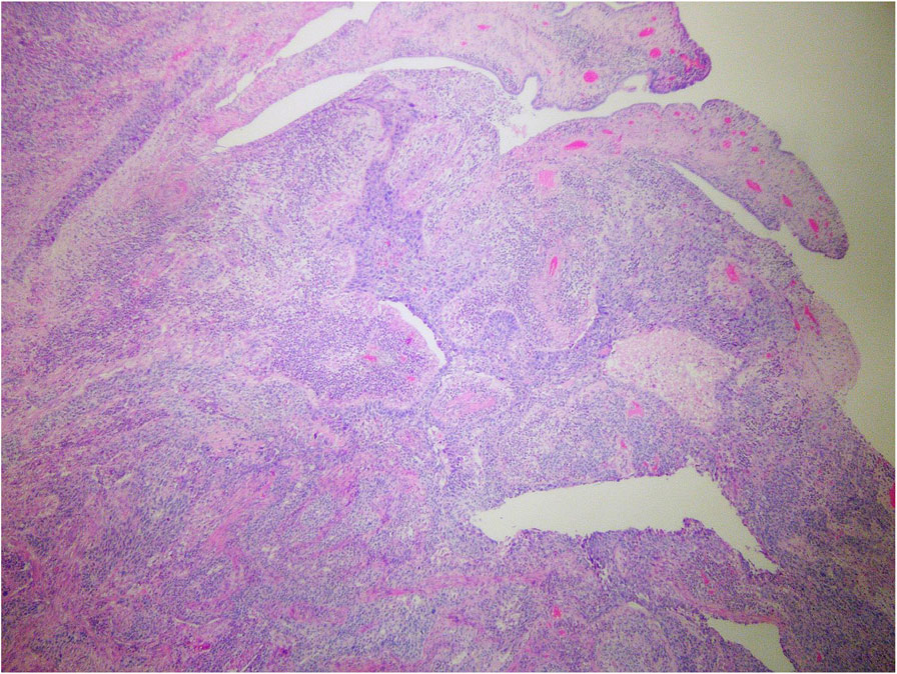
Metastasis of poorly differentiated carcinoma into the fimbrial part of left Fallopian tube; H&E, 5x and 40x

**Fig. 4:**
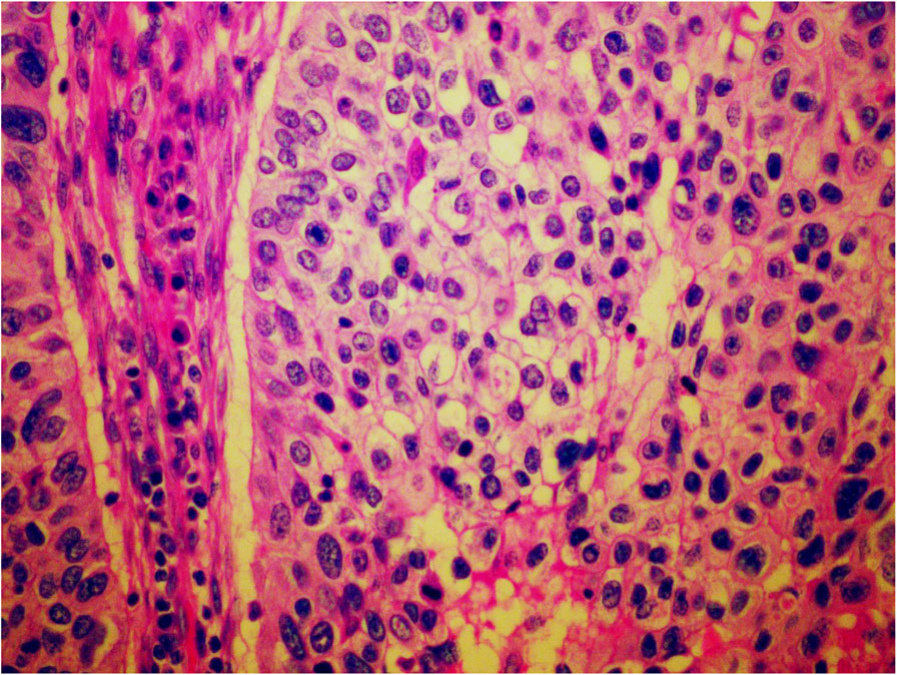
Metastasis of poorly differentiated carcinoma into the fimbrial part of left Fallopian tube; H&E, 5x and 40x

Genetic testing for germline variants was performed in April 2016 at our institut with next generation sequencing (NGS) and showed mutation in *BRCA2* gene: c.8755-1G > A, heterozygotic, which is currently classified as a pathogenic variant. From the whole blood, DNA was extracted using InnuPREP Master Blood kit (Analytik Jena, Thuringia, D). The coding sequence and exon/intron boundaries on DNA isolated from blood were enriched using Nextera DNA Library Preparation Kit in combination with TruSight Cancer Panel (Illumina, San Diego, USA), according to manufacturer’s protocol. NGS was performed on Illumina MiSeqDx Sequencing System (Illumina). Read alignment and variant calling was performed using MiSeq Reporter software 2.5.1. Variant annotation was performed using Variant Studio software 3.0 (Illumina) and Alamut Visual software 2.11 (Interactive Biosoftware, Rouen, France). Direct Sanger sequencing was performed to confirm mutations detected by NGS. For direct DNA sequencing, the samples were bidirectionally sequenced on an automated ABI 3500 genetic analyzer (Applied Biosystems, Foster City, CA). While still receiving adjuvant hormonal treatment, a mass in the left lower abdomen was found, measuring 6 × 5 cm. The Ca 15–3 level was elevated for the first time (35 kU/l, normal level below 30 kU/l), while the Ca 125 level was normal. Fine needle aspiration sampling was performed twice and revealed only poorly differentiated carcinoma; immunocytochemmistry could not have been done due to the lack of material. The tumor board decided for surgical removal of the lesion as if it was a primary ovarian cancer. She underwent surgery in June 2016, combining the risk-reducing (due to known *BRCA2* pathogenic variant) and primary ovarian cancer approach - total hysterectomy with bilateral adnexectomy and removal of the regional lymph nodes. The final histological report identified a metastasis of a poorly differentiated carcinoma, which was CK7 and GATA3 positive, and CK20, PAX-8, WT1 negative. ER and PgR as well as Her-2 receptor status were negative. It was clearly concluded that is a metastasis of a breast carcinoma in retroperitoneal lymph nodes as well as in the fimbrial part of the left Fallopian tube. Metastasis in the fimbrial part of the left Fallopian tube are seen in Figs. 3 and 4. Five out of 11 surgically removed lymph nodes were positive for malignancy. Since metastases of the breast cancer were triple-negative, she received additional 6 cycles of EC chemotherapy (epirubicin, cyclophosphamide). During chemotherapy her Ca 15–3 level returned to normal.

For the purpose of this article, the molecular analyses of the tumor tissue were done to find out whether this has been the same tumour all along. The DNA samples extracted from formalin-fixed paraffin embedded tumor tissue (FFPE) were used. DNA was extracted using GeneRead DNA FFPE Kit (QiagenGmbH, Hilden, Germany) from manually macro-dissected areas annotated by a pathologist by scraping directly off unstained standard glass slides (10 μm). Hematoxylin-eosin staining of the first sectioned slide was performed to visualize the presence of tumour cells, and to guide macro-dissection on unstained duplicate slides and to determine the area of the tissue cores. The coding sequence and exon/intron boundaries on DNA isolated from FFPE tumor tissue were enriched using TruSight Tumor 170 kit – TST 170 (Illumina, San Diego, USA) according to manufacturer’s protocol. Before the library preparation the DNA quality and quantity was assessed using Infinium FFPE QC and DNA Restoration Kit (Illumina). NGS was performed on Illumina NextSeq 550 (Illumina). Read alignment and variant calling was performed using Local App TruSight Tumor 170 v.1 software and Enrichment App on BaseSpase (Illumina). Variant annotation was performed using Variant Studio software 3.0 and Alamut Visual software 2.11 (Interactive Biosoftware).

The number of nonsynonymous somatic variants within all coding regions of the genes included in the panel is detected and quantified as number of variants per megabase. Since the total size of the regions sequenced with TST 170 is 533 kb (approximately 0.5 Mb) the detected number of somatic variants was multiplied by two. Therefore, the detected and quantified number of variants per megabase was in sample 01 (tumor from 1998) zero, in sample 02 (tumor from 2011) two, and in sample 03 (tumor from 2016) ten (Table 1). None of somatic variants had the tested samples in common, therefore alluding that all three tumors were of primary origin (Table 1).

**Table 1:** Variants detected in three different tumor samples of the same patient. In the table are included only variants reported in whole GnomAD population (ALL) or European or Non-Finish population (NFE) with frequencies < 1%. Variants classified as benign in ClinVar database were removed from the report

Gene	Variant type	cHGVS	pHGVS	AF (%) in sample 01	AF (%) in sample 02	AF (%) in sample 03	variant status (germline/somatic)	Frequencies in different population in GnomAD database
BRCA2	missense_variant	NM_000059.3:c.978C > A	NP_000050.2:p.(Ser326Arg)	16.96	24.69	15.98	confirmed germline variant^a^	ALL:0.090% - NFE:0.14%
FGF5	3_prime_UTR_variant	NM_004464.3:c.*1139 T > C		39.17	40.88	81.8	likely germline variant^b^	ALL:0.89% - NFE:0.82%
FGFR2	missense_variant	NM_000141.4:c.170C > T	NP_000132.3:p.(Ser57Leu)	40.64	50	88.62	likely germline variant^b^	ALL:0.45% - NFE:0.31%
STK11	synonymous_variant	NM_000455.4:c.945G > A	NM_000455.4:p.(Pro315=)	70.77	40.5	80.77	confirmed germline variant^a^	ALL:0.072% - NFE:0.073%
BCL6	splice_region_variant,intron_variant	NM_001706.4:c.1356-3 T > C	p.?	48.3	30.6	42.9	likely germline variant^b^	ALL:0.24% -NFE:0.18%
BRCA2	splice_acceptor_variant	NM_000059.3:c.8755-1G > A	p.?	72.3	72.6	81.2	confirmed germline variant^a^	Not known to gnomAD
AR	synonymous_variant	NM_000044.3:c.1365 T > G	NM_000044.3:p.(Gly455=)	13.82	nd	nd	somatic variant^c^	Not known to gnomAD
RPS6KB1	synonymous_variant	NM_001272043.1:c.621G > A	NM_001272043.1:p.(Gly207=)	8.99	nd	nd	somatic variant^c^	Not known to gnomAD
PDGFRB	missense_variant	NM_002609.3:c.1312G > T	NP_002600.1:p.(Gly438Cys)	nd	33.2	nd	somatic variant^c^	Not known to gnomAD
CTNNB1	synonymous_variant	NM_001904.3:c.765C > A	NM_001904.3:p.(Ala255=)	nd	nd	77.66	somatic variant^c^	Not known to gnomAD
ERBB4	missense_variant	NM_005235.2:c.670C > A	NP_005226.1:p.(Pro224Thr)	nd	nd	23.68	somatic variant^c^	Not known to gnomAD
FGF14	missense_variant	NM_175929.2:c.4G > A	NP_787125.1:p.(Val2Ile)	nd	nd	37.15	somatic variant^c^	ALL:0.00040%
NF1	missense_variant	NM_000267.3:c.7086C > G	NP_000258.1:p.(Asn2362Lys)	nd	nd	34.25	somatic variant^c^	Not known to gnomAD
TP53	missense_variant	NM_000546.5:c.844C > T	NP_000537.3:p.(Arg282Trp)	nd	nd	73.6	somatic variant^c^	ALL:0.00040% - NFE:0.00090%
TSC1	missense_variant	NM_000368.4:c.967C > A	NP_000359.1:p.(Pro323Thr)	nd	nd	32.35	somatic variant^c^	ALL:0.00040% - NFE:0.00090%

*AF* allele frequency of nucleotide variant in the sample, *nd* not detected, *cHGVS* variant description on coding DNA reference sequence according to Human Genome Variation Society, *pHGVS* variant description on protein reference sequence according to Human Genome Variation Society

^a^the variant was confirmed on DNA isolated from patient blood sample, ^b^variant was classified as likely gemline if the frequency in populations reported in GnomAD database were > 0,005, ^c^variants not reported in GnomAD or reported in GnomAD with the frequency < 0,005 in different populations

The patient, however continues her regular follow-up and is in complete remission at the time of writing this article (40 months after last chemotherapy).

## Discussion

We present an interesting case of a breast cancer metastasis into the fimbrial part of the Fallopian tube after bilateral breast carcinoma treatment in a patient with known *BRCA2* germline pathogenic variant.

Ovarian metastases are seen in different settings. A predilection site for metastasis is described in the past few years as metastatic organotropism [7, 8]. While in breast cancer there are several molecular and genetic patterns already associated with metastasizing to the brain, lungs, and bones [8, 9], yet to the best of our knowledge, there have been no markers that predict metastasis to the uterine adnexa identified so far. At 15% of the all ovarian metastases, primary tumor remains unknown [10]. In a Dutch population study, 14.3% of ovarian metastases were due to primary breast cancer, of which more than half were bilateral [10]. A group in Athens performed a 10 year review of the metastatic neoplasms to the ovary and found 15.4% of the neoplasms originating from primary breast cancer [11]. Hungarian group reported breast cancer to be the primary site in 20% [12]. A review of literature by Kubeček et al. [7] shows that breast is the primary site in 1.8% up to 33% of cases of ovarian metastases. Since the survival of patients with metastases into ovaries is worse than the survival of those with primary ovarian cancer, the distinction between the two is critically important to understand [6].

Looking from the other point of view, *BRCA2* carriers are more likely to develop a metachronous ovarian cancer than the general population. An Italian group analyzed risk-reducing surgery specimens in the 18 years observation period for either *BRCA1* and *BRCA2* carriers, non carriers or patients with unknown *BRCA1* and *BRCA2* status. 75% of women had a history of breast cancer and when performing a risk-reducing salpingo-oophorectomy, 3.6% of patients had an occult cancer, while only two out of 411 patients had a breast cancer metastasis in the uterine adnexa, alluding that a metastasis in the fimbrial part of the Fallopian tube is a rare event [3]. Rabban published a review with the comparison of histological features of primary ovarian cancer and breast cancer metastases into the uterine adnexa [4]. Only 1% of *BRCA1* and *BRCA2* positive patients in their series had a breast cancer metastasis into the uterine adnexa, again confirming the rarity of the event [4].. These data show that our case is interesting yet rare and not often described in the literature. However, late metastases in *BRCA2* positive patients are not uncommon. Regarding the interval from the first disease occurence in 1999 it is very unusual for a triple-negative breast cancer to have such a long and slow course. In the literature, there are reports of a receptor conversion through time [8]. The meta-analysis by Aurilio et al. showed that the rates of discordance of primary tumor and metastasis for ER and PgR were 20 and 33% [13]. They also noted that the conversion to negative receptor status at recurrence was seen more frequently than the positive conversion with rates of 24% vs. 14% for ER status and 46% vs. 15% for PgR status [13], which can be greatly attributed to the treatment given that select subclones with different phenotypes to emerge. For the purpose of this article a molecular analyses of all three samples were done using TST 170 gene panel and no common mutation was found. As this gene panel includes a limited set of genes, we cannot definitively exclude the possibility that the tumors are of the same origin. However, based on histological results, we conclude that it is more likely that there was a receptor status conversion over time due to hormonal treatment the patient was receiving after 2011 and that the metastasis in the fimbrial part of the left Fallopian tube was a metastasis of a 2011 tumour rather than a metastasis of a triple-negative cancer from 1998. Knowing that the conversion of receptor status is known to be associated with a worse prognosis [8], our patient is still in complete remission and continues her regular follow-up.

## Conclusions

This is an interesting case of *BRCA2* positive patient with bilateral primary breast cancer having a different receptor expression with a very long interval from primary disease occurrence and metastasis into the fimbrial part of the Fallopian tube. We conclude that there were two primary breast tumours and the one in 2011 spread into the fimbrial part of the Fallopian tube in 2016. Despite the fact that molecular analyses could not confirm the joint tumour origin, we believe that there was a receptor status conversion over time explaining different receptor status. However, after all the treatment received, the patient has a good quality of life and she is in complete remission at the time of writing this article.

## Data Availability

The dataset used during the current study are available from the corresponding author on reasonable request.

## Abbreviations

CMF: Cyclophosphamide, methotrexate and fluorouracil
DCIS: Ductal carcinoma in situ
EC: Epirubicin, cyclophosphamide
ER: Estrogen receptor
PgR: Progesteron receptor
MIB-1: Proliferation index
MRI: Magnetic resonance imaging

## References

[1] Zadnik V (2018). Cancer in Slovenia 2015.

[2] Paluch-Shimon S, Cardoso F, Sessa C, Balmana J, Cardoso MJ, Gilbert F, Senkus E, ESMO Guidelines Committee (2016). Prevention and screening in BRCA mutation carriers and other breast/ovarian hereditary cancer syndromes: ESMO clinical practice guidelines for cancer prevention and screening. Ann Oncol.

[3] Ricciardi E, Tomao F, Aletti G, Bazzurini L, Bocciolone L, Boveri S, Landoni F (2017). Risk-reducing Salpingo-oophorectomy in women at higher risk of ovarian and breast Cancer: a single institution prospective series. Anticancer Res.

[4] Rabban JT, Barnes M, Chen LM, Powell CB, Crawford B, Zaloudek CJ (2009). Ovarian pathology in risk-reducing salpingo-oophorectomies from women with BRCA mutations, emphasizing the differential diagnosis of occult primary and metastatic carcinoma. Am J Surg Pathol.

[5] Makris GM, Marinelis A, Battista MJ, Chrelias C, Papantoniou N (2017). An ovarian mass after breast cancer: Metachronous carcinoma or metastasis? A case report. Int J Surg Case Rep.

[6] Skirnisdottir I, Garmo H, Holmberg L (2007). Non-genital tract metastases to the ovaries presented as ovarian tumors in Sweden 1990-2003: occurrence, origin and survival compared to ovarian cancer. Gynecol Oncol.

[7] Kubeček O, Laco J, Špaček J, Petera J, Kopecký J, Kubečková A, Filip S (2017). The pathogenesis, diagnosis, and management of metastatic tumors to the ovary: a comprehensive review. Clin Exp Metastasis.

[8] Kimbung S, Loman N, Hedenfalk I (2015). Clinical and molecular complexity of breast cancer metastases. Semin Cancer Biol.

[9] Lorusso G, Ruegg C (2012). New insights into the mechanisms of organ-specific breast cancer metastasis. Semin Cancer Biol.

[10] Bruls J, Simons M, Overbeek LI, Bulten J, Massuger LF, Nagtegaal ID (2015). A national population-based study provides insight in the origin of malignancies metastatic to the ovary. Virchows Arch.

[11] Kondi-Pafiti A, Kairi-Vasilatou E, Iavazzo C, Dastamani C, Bakalianou K, Liapis A, Hassiakos D (2011). Metastatic neoplasms of the ovaries: a clinicopathological study of 97 cases. Arch Gynecol Obstet.

[12] Tamas J, Vereczkey I, Toth E (2015). Metastatic tumors in the ovary, difficulties of histologic diagnosis. Magy Onkol.

[13] Aurilio G, Disalvatore D, Pruneri G, Bagnardi V, Viale G, Curigliano G, Adamoli L (2014). A meta-analysis of oestrogen receptor, progesterone receptor and human epidermal growth factor receptor 2 discordance between primary breast cancer and metastases. Eur J Cancer.

